# The Underlying Mechanism Involved in Gefitinib Resistance and Corresponding Experiment Validation in Lung Cancer

**DOI:** 10.1155/2023/9658912

**Published:** 2023-05-09

**Authors:** Puwei Song, Jianghui Zhou, Kaiqin Wu, Wenli Wang, Shaorui Gu

**Affiliations:** ^1^Department of Thoracic Surgery, Shanghai Tongji Hospital, School of Medicine, Tongji University, Shanghai, China; ^2^Department of Cardiothoracic Surgery, Children's Hospital of Nanjing Medical University, Nanjing, China; ^3^Department of Thoracic Surgery, Northern Jiangsu People's Hospital Affiliated to Yangzhou University, Yangzhou, China

## Abstract

**Background:**

Gefitinib resistance remains a major problem in the treatment of lung cancer. However, the underlying mechanisms involved in gefitinib resistance are largely unclear.

**Methods:**

Open-accessed data of lung cancer patients were downloaded from The Cancer Genome Atlas Program and Gene Expression Omnibus databases. CCK8, colony formation, and 5-ethynyl-2′-deoxyuridine assays were utilized to evaluate the cell proliferation ability. Transwell and wound-healing assays were utilized to evaluate the cell invasion and migration ability. Quantitative real-time PCR was utilized to detect the RNA level of specific genes.

**Results:**

Here, we obtained the expression profile data of wild and gefitinib-resistant cells. Combined with the data from the TCGA and GDSC databases, we identified six genes, RNF150, FAT3, ANKRD33, AFF3, CDH2, and BEX1, that were involved in gefitinib resistance in both cell and tissue levels. We found that most of these genes were expressed in the fibroblast of the NSCLC microenvironment. Hence, we also comprehensively investigated the role of fibroblast in the NSCLC microenvironment, including its biological effect and cell interaction. Ultimately, CDH2 was selected for further analysis for its prognosis correlation. In vitro experiments presented the cancer-promoting role of CDH2 in NSCLC. Moreover, cell viability detection showed that the inhibition of CDH2 could significantly decrease the IC50 of gefitinib in NSCLC cells. GSEA showed that CDH2 could significantly affect the pathway activity of PI3K/AKT/mTOR signaling.

**Conclusions:**

This study is aimed at investigating the underlying mechanism involved in gefitinib resistance to lung cancer. Our research has improved researchers' understanding of gefitinib resistance. Meanwhile, we found that CDH2 could lead to gefitinib resistance through PI3K/AKT/mTOR signaling.

## 1. Introduction

Lung cancer remains the most prevalent and dangerous malignant tumor worldwide, resulting in over one million related death cases per year [1]. Lung cancer is a multifactorial disease, and its specific mechanism is still unclear, but current research has found that the incidence of lung cancer is often related to environmental factors, lifestyle, genomic differences, and so on [2]. For now, patients at the early stage of the disease can often obtain long-term treatment benefits and a satisfactory prognosis from radical surgery [3]. Unfortunately, many lung cancer patients have already reached the late stages of the disease when they are diagnosed [4]. Lung cancers that have advanced are mostly treated with chemotherapy. Nonetheless, the effectiveness of chemotherapy is often limited, coupled with cytotoxicity and side effects, which exacerbate the patients' medical burden and quality of life [5].

Gefitinib is suitable for the treatment of locally advanced or metastatic non-small cell lung cancer (NSCLC) that has received chemotherapy or is not appropriate for chemotherapy [6]. Gefitinib can effectively improve the prognosis of patients with advanced NSCLC, and it has also been reported that when combined with chemotherapy, gefitinib can improve the therapy effect on lung cancer patients [7]. In clinical application, gefitinib may have acquired drug resistance, thus reducing its therapeutic effect [8]. Research has begun to focus on the biological mechanisms involved in gefitinib resistance. Chen et al. noticed that the lncRNA CASC9 could affect gefitinib resistance by epigenetically suppressing DUSP1 [9]. Liu et al. found that METTL3, an m6A methyltransferase, could regulate the gefitinib resistance by inducing autophagy and affecting *β*-elemene [10]. Cheng and Tong revealed that in NSCLC, the interaction between FLNA and ANXA2 could lead to the resistance of gefitinib through activating Wnt signaling [11]. Hence, exploring the factors influencing gefitinib resistance from the internal biological mechanism of tumors can provide a prospective reference for clinical application.

Here, we obtained the expression profile data of wild and gefitinib-resistant cells. Combined with the data from the TCGA and GDSC databases, we identified six genes, RNF150, FAT3, ANKRD33, AFF3, CDH2, and BEX1, that were involved in gefitinib resistance in both cell and tissue levels. We found that most of these genes were expressed in the fibroblast of the NSCLC microenvironment. Hence, we also comprehensively investigated the role of fibroblast in the NSCLC microenvironment, including its biological effect and cell interaction. Ultimately, CDH2 was selected for further analysis for its prognosis correlation. In vitro experiments presented the cancer-promoting role of CDH2 in NSCLC. Moreover, cell viability detection showed that the inhibition of CDH2 could significantly decrease the IC50 of gefitinib in NSCLC cells. We noticed that CDH2 could lead to gefitinib resistance through PI3K/AKT/mTOR signaling.

## 2. Methods

### 2.1. Download and Collection of Public Data from The Cancer Genome Atlas Program (TCGA)

The TCGA database provides the gene expression data and clinical information of NSCLC patients, which was downloaded for the analysis (524 samples from TCGA-LUAD and 503 samples from TCGA-LUSC). The initial gene expression data of a single sample was in STAR counts format and was summarized in R language and converted to TPM. Clinical data are organized by a Perl script written by the author. Before data analysis, data preprocessing is used to improve data quality. Its brief process includes annotation of the ENSG id, data standardization, and log2 conversion. The IC50 data of gefitinib was obtained from the Genomics of Drug Sensitivity in Cancer (GDSC) database [12].

### 2.2. Public Data from Gene Expression Omnibus (GEO) Database

The GSE123066 project was selected, and its data was obtained from the GEO database. GSE123066 provides the total RNA data sequenced from wild and gefitinib-resistant NSCLC cell lines. Data were directly downloaded from the “Series Matrix File(s)” link. Further patient information including gender, age, stage, and survival data is provided in Supplemental Table 1.

### 2.3. Differentially Expression Gene (DEGs) Analysis

We used the limma package for DEG analysis based on the detailed threshold [13].

### 2.4. Investigation of the Biological Aspect

Gene Ontology (GO) and Kyoto Encyclopedia of Genes and Genomes (KEGG) were conducted for biological investigation [14]. Gene set enrichment analysis (GSEA) was utilized to identify the biological differences between the two groups based on the hallmark pathway set [15].

### 2.5. Prognosis Evaluation

The evaluation of patient prognosis was completed using the Kaplan-Meier (KM) survival curves.

### 2.6. Single-Cell Analysis

The expression pattern of specific genes at the single-cell level and potential cell interactions was evaluated using the TSICH [16].

### 2.7. Cell Culture

The cell lines used include BEAS-2B, H441, H1299, and A549. All these cells were cultured in the DMEM culture medium under standard conditions.

### 2.8. Quantitative Real-Time PCR (qRT-PCR)

The whole process of qRT-PCR was conducted following the standard protocol [17]. The primers used were as follows: CDH2, forward primer, 5′-TCAGGCGTCTGTAGAGGCTT-3′, reverse primer, 5′-ATGCACATCCTTCGATAAGACTG-3′.

### 2.9. Cell Transfection

The whole process of cell transfection was conducted following the standard protocol [17]. The sh-CDH2 and control plasmids were obtained from Genechem, Shanghai, China.

### 2.10. Cell Proliferation Assays

Cell proliferation ability was evaluated using the CCK8, colony formation, and 5-ethynyl-2′-deoxyuridine (EdU) assays. The whole process of qRT-PCR was conducted following the standard protocol [18, 19].

### 2.11. Transwell Assay

The whole process of transwell assay was conducted following the standard protocol [20].

### 2.12. Wound-Healing Assay

The whole process of the wound-healing assay was conducted following the standard protocol [20].

### 2.13. Detection of Cell Viability

The whole process of cell viability detection was conducted following the standard protocol [18].

### 2.14. Statistical Analysis

The statistical analysis was conducted in R, GraphPad Prism 8, and SPSS software. The 0.05 was set as the statistical threshold. For the comparison of two groups using Wilcoxon's rank-sum tests and the comparison between continuous variables using Wilcoxon's rank-sum tests, Wilcoxon's test was used to examine statistical significance.

## 3. Results

### 3.1. Identification of the Genes Contributing to Gefitinib Resistance and Their Biological Role

**Figure 1:**
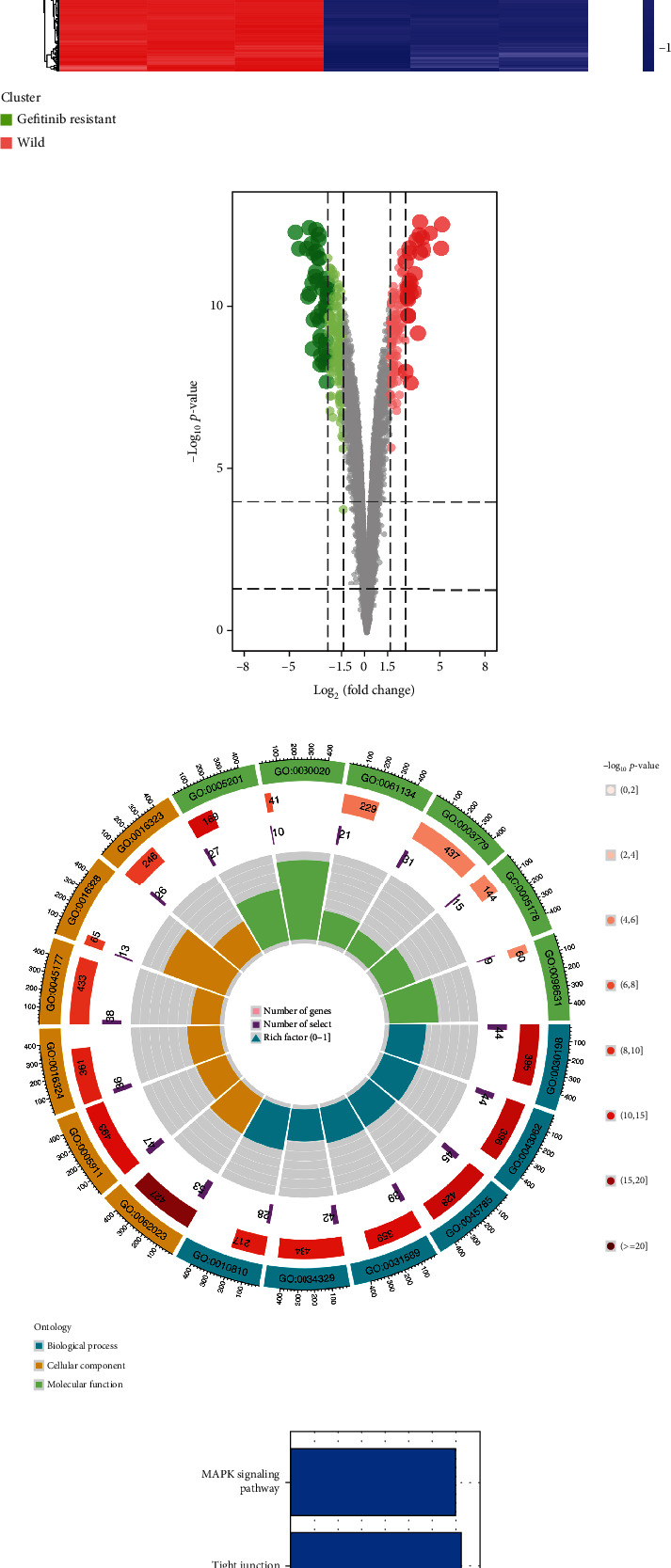
Collection of the molecules involved in gefitinib resistance. Notes: (a) Heatmap was used to present the expression pattern of DEGs between the wild and gefitinib-resistant NSCLC cell lines. (b) 476 downregulated and 322 upregulated molecules were identified as involved in the gefitinib resistance in the cell level. (c) GO analysis of these DEGs. (d) KEGG analysis of these DEGs.

Through the limma package, we identified the DEGs between the wild and gefitinib-resistant NSCLC cell lines, which are shown in Figure 1(a). Totally, 476 downregulated and 322 upregulated molecules were identified as involved in the gefitinib resistance in the cell level (Figure 1(b)). GO analysis revealed that these DEGs were mainly associated with GO:0005201, GO:0030020, GO:0061134, GO:0003779, GO:0005178, GO:0098631, GO:0030198, GO:0043062, GO:0045785, GO:0031589, GO:0034329, GO:0010810, GO:0062023, GO:0005911, GO:0016324, GO:0045177, GO:0016328, and GO:0016323 (Figure 1(c)), and all the results of GO analysis were provided in Supplemental Table 2. KEGG analysis indicated that these DEGs were primarily enriched in the MAPK signaling pathway, PI3K/AKT signaling pathway, leukocyte transendothelial migration, cell adhesion molecules, and tight junction (Figure 1(d)).

### 3.2. RNF150, FAT3, ANKRD33, AFF3, CDH2, and BEX1 Were Correlated with Gefitinib Resistance in Both Cell and Tissue Levels

**Figure 2:**
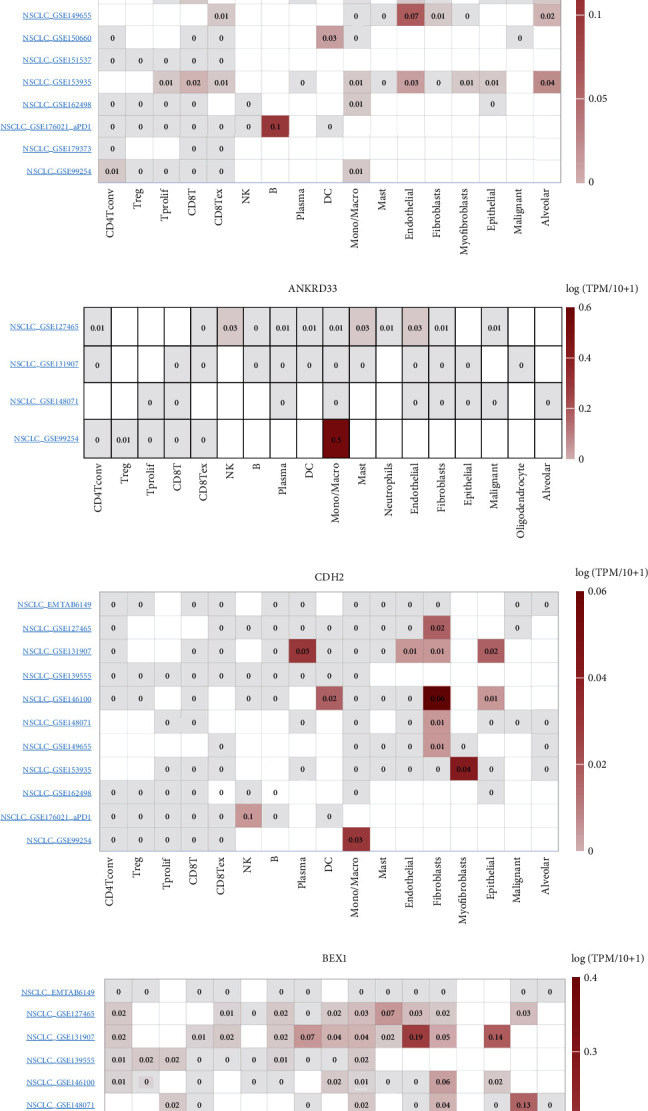
Identification of the hub genes. Notes: (a) DEG analysis between the top 50 patients with the highest or the lowest IC50 (LUAD). (b) DEG analysis between the top 50 patients with the highest or the lowest IC50 (LUSC). (c) The intersection of GSE123066, TCGA-LUAD, and TCGA-LUAD identified six genes. (d) The expression level of RNF150, FAT3, ANKRD33, AFF3, CDH2, and BEX1 in wild and gefitinib resistance cells. (e–j) The single-cell level of RNF150, FAT3, ANKRD33, AFF3, CDH2, and BEX1.

We next obtained the IC50 data of gefitinib in the GDSC database (lung cancer). Then, we performed a DEG analysis between the top 50 patients with the highest or lowest IC50. Finally, 1711 upregulated genes were identified in LUAD (Figure 2(a)) and 2302 upregulated genes were identified in LUSC (Figure 2(b)). The intersection of GSE123066, TCGA-LUAD, and TCGA-LUAD identified six genes, RNF150, FAT3, ANKRD33, AFF3, CDH2, and BEX1, indicating that these genes were involved in gefitinib resistance in both cell and tissue levels (Figure 2(c)). Results indicated that all these genes were overexpressed in the gefitinib-resistant cells (Figure 2(d)). Single-cell analysis revealed that AFF3 was primarily expressed in B and endothelial cells (Figure 2(e)); ANKRD33 was primarily expressed in mono/macrocells (Figure 2(f)); CDH2 was primarily expressed in fibroblast cells (Figure 2(g)); BEX1 was primarily expressed in mono/macrocells (Figure 2(h)); FAT3 was primarily expressed in mono/macro and fibroblast cells (Figure 2(i)); RNF150 was primarily expressed in fibroblast l cells (Figure 2(j)).

### 3.3. Role of Fibroblast in NSCLC Microenvironment

**Figure 3:**
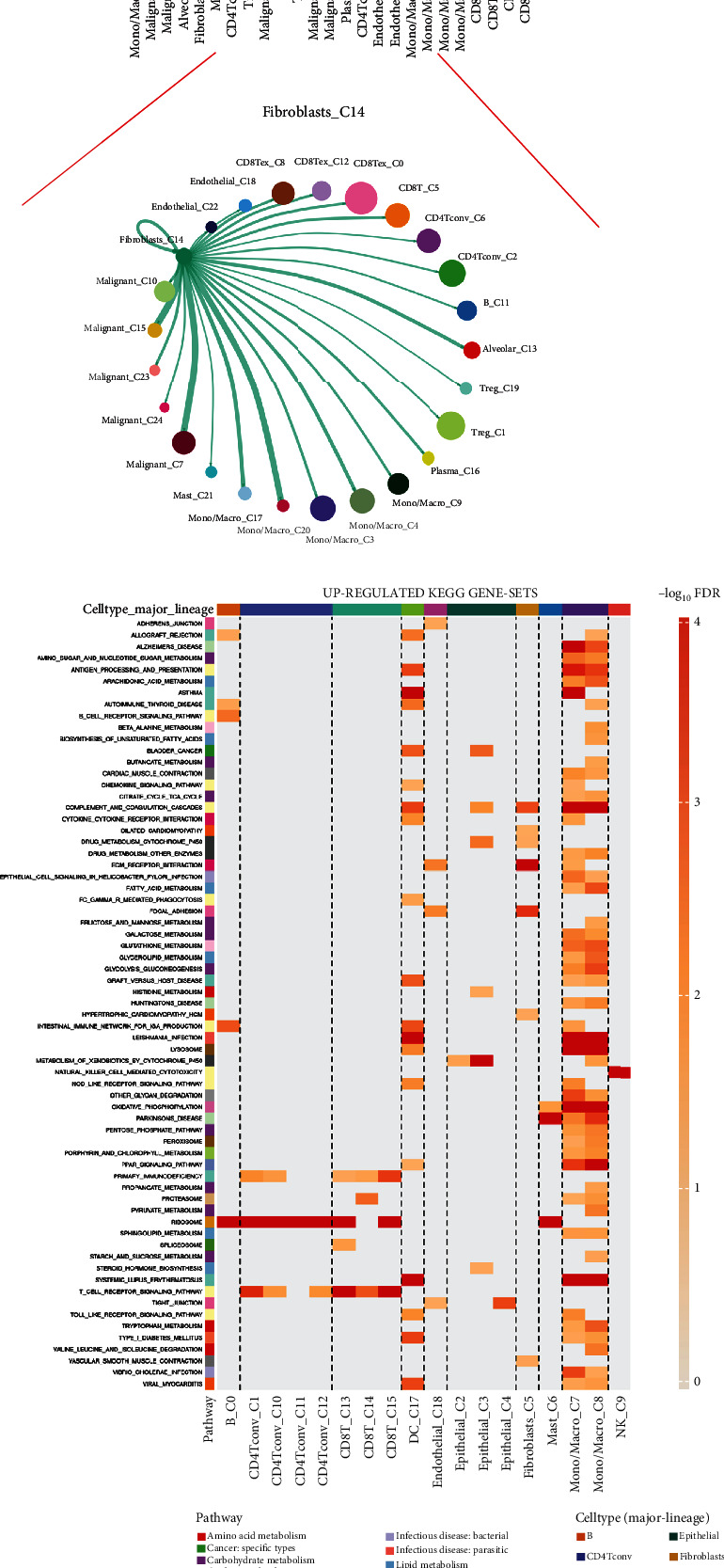
Role of CAFs in NSCLC microenvironment. Notes: (a) The upregulated KEGG terms regulated by CAFs (EMTAB-6149 cohort). (b) The downregulated KEGG terms regulated by CAFs (EMTAB-6149 cohort). (c) The upregulated hallmark terms regulated by CAFs (EMTAB-6149 cohort). (d) The downregulated KEGG terms regulated by CAFs (EMTAB-6149 cohort). (e) Cell interaction in EMTAB-6149 cohort. (f) The upregulated KEGG terms regulated by CAFs (LUAD-GSE146100 cohort). (g) The downregulated KEGG terms regulated by CAFs (LUAD-GSE146100 cohort). (h) The upregulated hallmark terms regulated by CAFs (LUAD-GSE146100 cohort). (i) The downregulated KEGG terms regulated by CAFs (LUAD-GSE146100 cohort). (j) Cell interaction in EMTAB-6149 cohort.

Considering that most of these six genes were expressed in fibroblast, following this, we investigated the role of fibroblasts in the NSCLC microenvironment. In the EMTAB-6149 cohort, we found that in KEGG analysis, fibroblast was correlated with upregulated focal adhesion, ECM receptor interaction, dilated cardiomyopathy, and B cell receptor signaling pathway while downregulating ribosome, cell adhesion molecules (CAMs), leishmania infection, and some immune-related pathway activities (Figures 3(a) and 3(b)). As for the hallmark pathway, we noticed that fibroblast was positively correlated with UV response DN, adipogenesis, epithelial_mesenchymal_transition (EMT), angiogenesis, myogenesis, coagulation, and hypoxia (Figure 3(c)), while negatively correlated with mTORC signaling, E2F targets, allograft rejection, and the interferon alpha response (Figure 3(d)). Cell interaction analysis showed that the fibroblast could interact with malignant and endothelial cells (Figure 3(e)). In the LUAD-GSE146100 cohort, fibroblasts were positively correlated with focal adhesion, ECM receptor interaction, dilated cardiomyopathy, complement, and coagulation cascades while negatively correlated with many immune-related pathways (Figures 3(f) and 3(g)). For hallmark analysis, fibroblasts were positively correlated with angiogenesis, apical junction, apoptosis, coagulation, EMT, myogenesis, and UV response DN, yet negatively correlated with allograft rejection, IL2/STAT5 signaling, complement, mTORC1 signaling, and PI3K/AKT/mTOR signaling (Figures 3(h) and 3(i)). Cell interaction analysis indicated that in the LUAD-GSE146100 cohort, fibroblasts mainly interacted with epithelial and endothelial cells (Figure 3(j)).

### 3.4. Prognosis Analysis of RNF150, FAT3, ANKRD33, AFF3, CDH2, and BEX1

**Figure 4:**
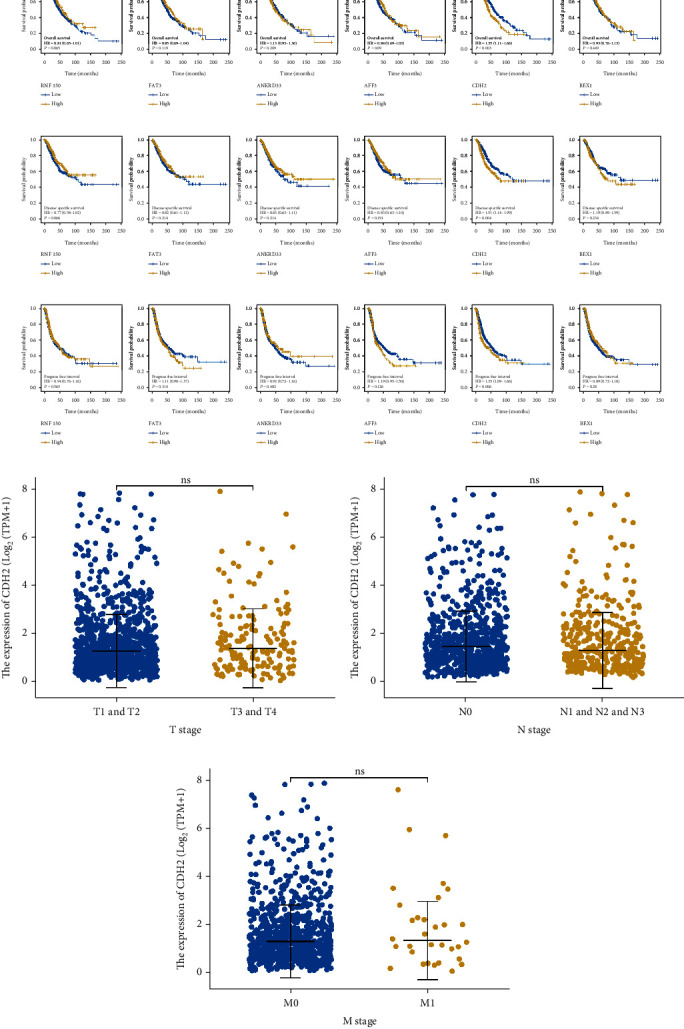
Prognosis analysis. Notes: (a) Performance of overall survival of RNF150, FAT3, ANKRD33, AFF3, CDH2, and BEX1. (b) Performance of disease free survival of RNF150, FAT3, ANKRD33, AFF3, CDH2, and BEX1. (c) Performance of progression free survival of RNF150, FAT3, ANKRD33, AFF3, CDH2, and BEX1. (d–f) Expression level of CDH2 in patients with different clinical features.

Then, we tried to explore the clinical value of RNF150, FAT3, ANKRD33, AFF3, CDH2, and BEX1. KM survival curves indicated that only CDH2 significantly affects patient survival (Figure 4(a), overall survival, HR = 1.35; Figure 4(b), disease-free survival, HR = 1.51; Figure 4(c), progression-free survival, HR = 1.35). However, no significant difference was found in patients with better or worse clinical features (Figures 4(d)–4(f)).

### 3.5. CDH2 Facilitates the Malignant Biological Behaviors of NSCLC Cells

**Figure 5:**
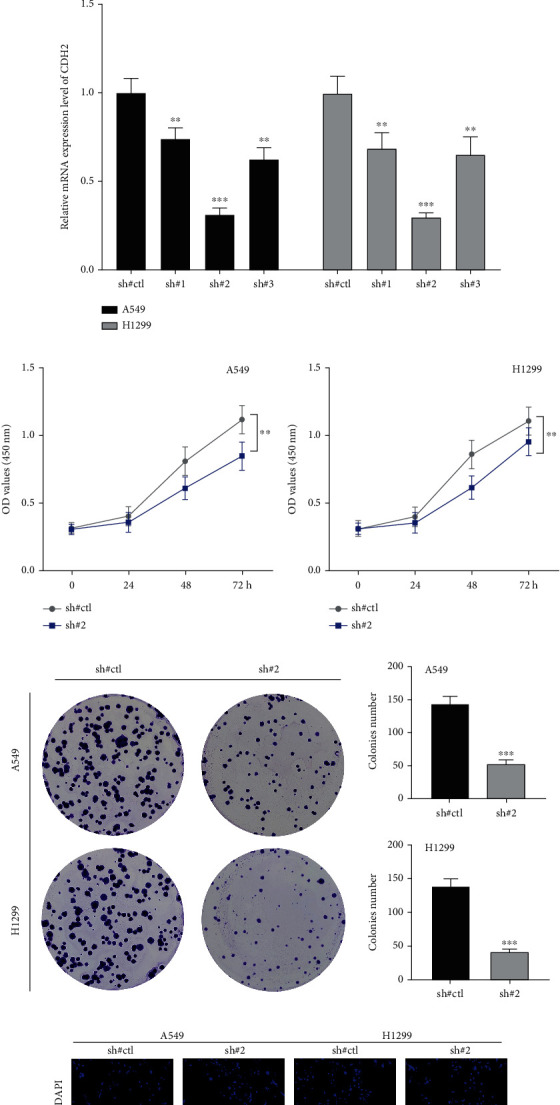
CDH2 promotes the cell proliferation of NSCLC. Notes: (a) The expression level of CDH2 in paired NSCLC tissue. (b) Expression level of CDH2 in NSCLC cells. (c) Knockdown efficiency of CDH2. (d, e) CCK8 assay was performed in sh-CDH2 and control cells. (f) Colony formation assay was performed in sh-CDH2 and control cells. (g) EdU assay was performed in sh-CDH2 and control cells.

**Figure 6:**
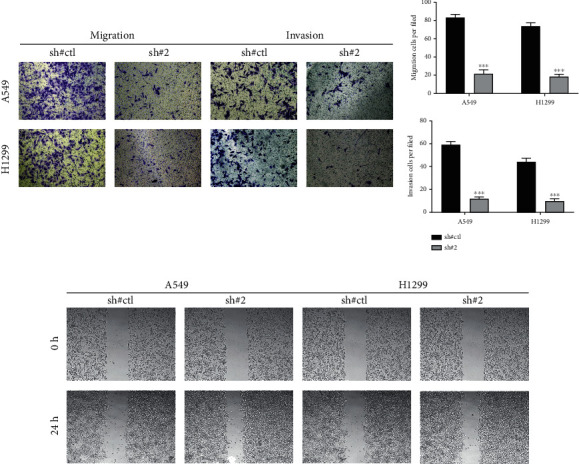
CDH2 facilitates the invasion and migration of NSCLC cells. Notes: (a) Transwell assay was performed in sh-CDH2 and control cells. (b) Wound-healing assay was performed in sh-CDH2 and control cells.

We next investigated the influence of CDH2 on NSCLC biological behaviors. Data of TCGA indicated that CDH2 was overexpressed in NSCLC tissue (Figure 5(a)). Also, in the cell level, the NSCLC cell lines had a higher CDH2 expression compared to the normal cell line (Figure 5(b)). The result of qRT-PCR demonstrated that the knockdown efficiency of sh#2 might have the best performance, therefore it was selected for further analysis (Figure 5(c)). The CCK8 assay indicated that the suppression of CDH2 in NSCLC cells could inhibit the proliferation ability (Figures 5(d) and 5(e)). The same result was also observed through colony formation and EdU assay (Figures 5(f) and 5(g)). A transwell assay was applied to evaluate the invasion and migration abilities of NSCLC cells. Results indicated that the inhibition of CDH2 could remarkably reduce the invasion and migration cells per filled compared to the control group (Figure 6(a)). The wound-healing assay showed that the inhibition of CDH2 could significantly hamper the cell mobility of NSCLC cells (Figure 6(b)).

### 3.6. CDH2 Lead to Gefitinib Resistance through PI3K/AKT/mTOR Signaling

**Figure 7:**
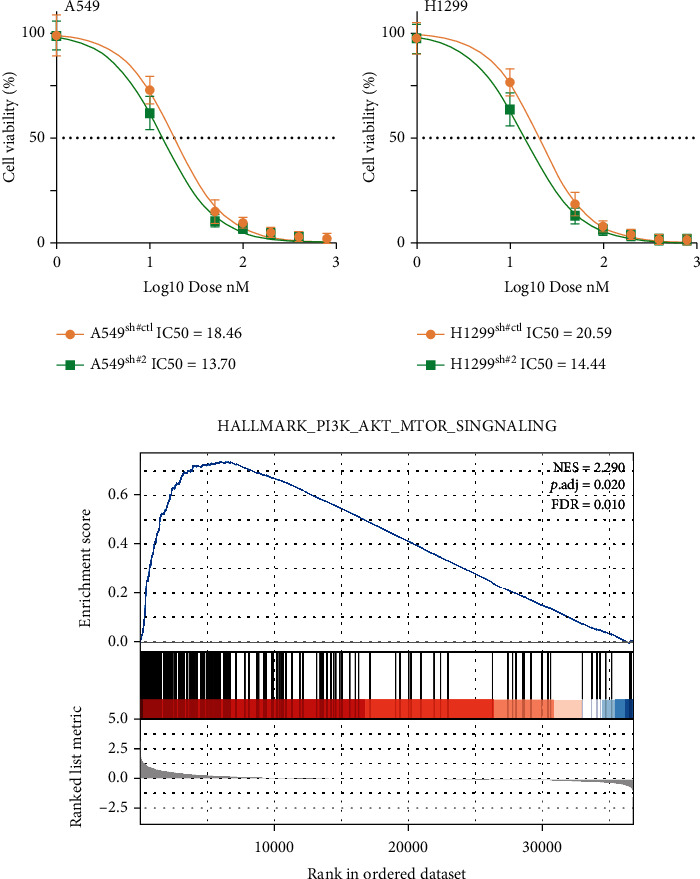
CDH2 could lead to gefitinib resistance through PI3K/AKT/mTOR signaling. Notes: (a, b) Cell viability detection in sh-CDH2 and control cells. (c) GSEA showed that CDH2 significantly affect the pathway activity of PI3K/AKT/mTOR signaling.

We next added gefitinib to the CDH2-inhibited and control cells. Cell viability detection showed that the inhibition of CDH2 could significantly decrease the IC50 of gefitinib in both A549 and H1299 cells (Figure 7(a): A549, sh#ctl, IC50 = 18.46, sh#2, IC50 = 13.70; Figure 7(b): H1299, sh#ctl, IC50 = 18.46, sh#2, IC50 = 13.70). GSEA showed that CDH2 could significantly affect the pathway activity of PI3K/AKT/mTOR signaling (Figure 7(c)). The previous study indicated that the PI3K/AKT/mTOR pathway could affect the gefitinib resistance. Therefore, we tried to evaluate whether CDH2 could affect the pathway activity of PI3K/AKT/mTOR signaling.

## 4. Discussion

Although the reform of medical technology has brought high-quality medical services, lung cancer is still facing the threat of a high incidence rate and mortality [21]. In clinical practice, early detection often enables patients to undergo radical surgery at the early stage of the disease and obtain long-term treatment benefits [22]. However, since the early symptoms are not obvious, many lung cancer patients have already had disease progression at the time of initial diagnosis and lost the best opportunity for surgery [23]. Gefitinib can improve the prognosis of advanced NSCLC patients, but it is still limited by acquired drug resistance. For several years now, a crucial role has been played in bioinformatics analysis in cancer research [24–26]. The objective of this study was to determine the underlying mechanism behind lung cancer resistance to gefitinib through bioinformatics analysis and corresponding experiment validation.

Here, we obtained the expression profile data of wild and gefitinib-resistant cells. Combined with the data from the TCGA and GDSC databases, we identified six genes, RNF150, FAT3, ANKRD33, AFF3, CDH2, and BEX1, that were involved in gefitinib resistance in both cell and tissue levels. We found that most of these genes were expressed in the fibroblast of the NSCLC microenvironment. Hence, we also comprehensively investigated the role of fibroblast in the NSCLC microenvironment, including its biological effect and cell interaction. Ultimately, CDH2 was selected for further analysis for its prognosis correlation. In vitro experiments presented the cancer-promoting role of CDH2 in NSCLC. Moreover, cell viability detection showed that the inhibition of CDH2 could significantly decrease the IC50 of gefitinib.

We noticed that the DEGs were primarily enriched in the MAPK signaling pathway, PI3K/AKT signaling pathway, leukocyte transendothelial migration, cell adhesion molecules, and tight junction. Some previous studies have begun to explore the relationship between the above pathways and gefitinib. AlAsmari et al. revealed that MAPK/NF-*κ*B signaling could significantly lighten the cardiotoxicity induced by gefitinib [27]. Lu et al. found that in NSCLC, the trans-3,5,4′-trimethoxystilbene could inhibit the MAPK/Akt/Bcl-2 axis by upregulating miR-345 and miR-498, further reducing gefitinib resistance [28]. Zheng et al. found that polyphyllin II could regulate the gefitinib resistance by affecting the PI3K/Akt/mTOR signaling [29]. These results indicated that the activation of the above pathways may promote the development of gefitinib resistance under the influence of specific factors.

Our results also found that RNF150, FAT3, ANKRD33, AFF3, CDH2, and BEX1 were involved in gefitinib resistance in both cell and tissue levels. The role of these genes in cancer has also been reported. Guo et al. found that FAT3 was correlated with the prognosis of esophageal cancer patients [30]. In breast cancer, Shi et al. found that FAT3 was associated with resistance to tamoxifen [31]. Wang et al. revealed that the BEX1 methylation regulated by DNMT1 could contribute to liver cancer stemness and tumorigenicity [32]. Lee et al. found that BEX1 could promote glioblastoma progression by activating the YAP/TAZ signaling [33]. In NSCLC, cancer-associated fibroblasts might exert an important role. Yi et al. noticed that the CAFs could lead to EMT and the resistance of EGFR-TKI by mediating the HGF/IGF-1/ANXA2 signaling axis [34].

The arrival of the big data era has produced massive data. On this basis, the secondary analysis of open data or research can facilitate researchers and draw valuable conclusions. Based on high-quality data and analysis process, our research has improved researchers' understanding of gefitinib resistance. Nevertheless, some limitations should be noted. Firstly, most of the populations included in the study are from Western countries. There are biological differences between populations of different races, which may reduce the reliability of our conclusions. Secondly, some patients have incomplete clinical baseline data, which may lead to sample bias to some extent.

## Data Availability

The data that support the findings of this study are available from the corresponding authors upon reasonable request.

## Supplementary Materials

Supplementary 1 Supplemental Table 1: the detailed clinicopathological parameters of patient.

Supplementary 2 Supplemental Table 2: all the results of GO analysis of the genes contributing to gefitinib resistance.
