# Correction to “The Underlying Mechanism Involved in Gefitinib Resistance and Corresponding Experiment Validation in Lung Cancer”

**DOI:** 10.1155/mi/9865015

**Published:** 2026-04-25

P. Song, J. Zhou, K. Wu, W. Wang, and S. Gu, “The Underlying Mechanism Involved in Gefitinib Resistance and Corresponding Experiment Validation in Lung Cancer,” *Mediators of Inflammation* 2023, no. 1 (2023): 9658912, https://doi.org/10.1155/2023/9658912.

In the above article, Figure 6b was misassembled while preparing the composite figures. Specifically, the image depicting the scratch assay for the H1299 cell line with sh‐CDH2 0 h was duplicated with the version of the scratch assay for the H1299 cell line with sh‐Control 0 h. The correct Figure 6 is as follows:

Figure 6CDH2 facilitates the invasion and migration of NSCLC cells. Notes: (a) Transwell assay was performed in sh‐CDH2 and control cells. (b) Wound‐healing assay was performed in sh‐CDH2 and control cells.

**Figure:**
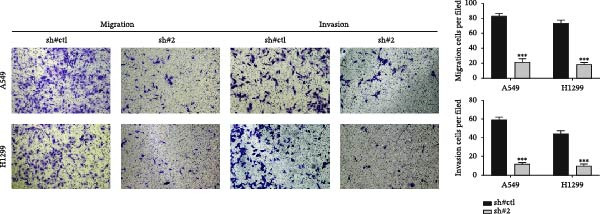
(a)

**Figure:**
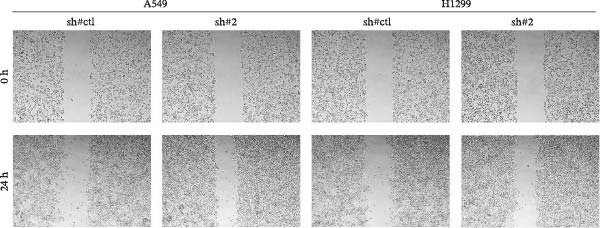
(b)

In addition, reference [26], “Zhang X., Zhang T., Ren X., Chen X., Wang S., and Qin C., Pyrethroids toxicity to male reproductive system and offspring as a function of oxidative stress induction: rodent studies, *Frontiers in Endocrinology* (2021) 12, article 656106, https://doi.org/10.3389/fendo.2021.656106, 34122335.” was incorrectly included.

Reference [26] should be: “Yang Q., Zhang H., Wei T., Lin A., Sun Y., Luo P., and Zhang J., “Single‐Cell RNA Sequencing Reveals the Heterogeneity of Tumor‐Associated Macrophages in Non‐Small Cell Lung Cancer and Differences Between Sexes,” *Frontiers in Immunology*, vol. 12, article 756722, 2021, https://doi.org/10.3389/fimmu.2021.756722.”

We apologize for these errors.

